# Unmasking of Sarcoidosis After Successful Management of Cushing's Syndrome

**DOI:** 10.7759/cureus.3896

**Published:** 2019-01-16

**Authors:** Brian D Noreña-Rengifo, Juan D Gomez-Corrales, Alejandro Roman-Gonzalez

**Affiliations:** 1 Radiology, Universidad De Antioquia, Medellín, COL; 2 Endocrinology, Hospital Universitario San Vicente Fundación, Medellín, COL; 3 Endocrinology, Universidad De Antioquia, Medellin, COL

**Keywords:** cushing’s syndrome, autoimmune disease, glucocorticoids, sarcoidosis

## Abstract

Cushing's syndrome is characterized by excessive glucocorticoid secretion leading to immunosuppression. The unmasking or aggravation of autoimmune diseases upon the normalization of cortisol levels after Cushing’s syndrome cure has been reported infrequently.

The case of a 45-year-old woman who presented with an 11-month history of severe signs and symptoms of hypercortisolism is reported. Hormonal tests suggested the presence of adrenocorticotropic hormone (ACTH)-independent Cushing's syndrome. Imaging studies detected an adrenal adenoma. The patient underwent laparoscopic adrenalectomy, and the mass was resected. Five months later the patient developed generalized arthralgias, malaise, a dry cough, and erythema nodosum. A diagnosis of sarcoidosis was confirmed by imaging and biopsy.

## Introduction

Cushing’s syndrome is a rare disease characterized by excessive cortisol secretion. Long-term complications of hypercortisolism are significant and include osteoporosis, hypertension, diabetes mellitus, hirsutism, and amenorrhea. In addition, severe hypercortisolism is associated with immunosuppression that predisposes to infections. Resolution of Cushing’s syndrome is associated with low or decreased cortisol levels. The low cortisol state may be associated with re-activation of the immune system and unmasking of autoimmune diseases. Sarcoidosis is a multisystem granulomatous disorder of unknown etiology that may involve any organ system and is characterized by the aggregation of T-lymphocytes, macrophages, and non-caseating granulomas leading to progressive inflammation and fibrosis [1]. Typical presentations of this disease affect the lungs, the eyes or the skin. Sarcoidosis is a potential immune disorder masked by severe hypercortisolism in Cushing syndrome. Here, we report a rare case of a patient who presented with sarcoidosis after surgical treatment of Cushing’s syndrome, highlighting the importance of adequate follow-up and the potential development of autoimmune or inflammatory diseases after the resolution of hypercortisolism.

## Case presentation

A 45-year-old female patient with a past medical history of hypertension and dyslipidemia presented with an 11-month history of numerous episodes of chest pain, palpitations, and dyspnea. These symptoms were associated with persistent edema of the hands, legs, and face. An ambulatory electrocardiogram and cardiac stress test were normal. The patient was treated for hypertension with enalapril 20 mg twice daily, hydrochlorothiazide 25 mg once daily and propranolol 20 mg twice daily. On physical examination, she had bilateral nonpainful mobile supraclavicular lymph nodes that were larger on the right side (1.5 cm), and a cushingoid habitus with abdominal striae, centripetal obesity, a full moon face, and a buffalo neck. Blood tests, including a complete blood count, electrolytes, albumin, renal function tests, and thyroid function test, were within the normal range. Laboratory evaluation for Cushing’s syndrome confirmed the diagnosis of an adrenocorticotropic-independent Cushing’s syndrome (Table 1).

**Table 1:** Main laboratory results. ACTH: Adrenocorticotropic hormone; UFC: Urinary-free cortisol.

Parameter	Result	Reference value
24-h UFC – outpatient (ug/l)	587.52	20-90
Cortisol 11 pm (ug/dL)	18	5-18
24-h UFC – inpatient (ug/l)	742	32-143
Serum cortisol after low-dose dexamethasone suppression test	20 ug/dl	<1.8 ug/dl
ACTH	<1 pg/ml

An abdominal computed tomography (CT) scan showed a 3.7-cm diameter right adrenal mass with clearly identifiable borders and soft tissue and fatty density consistent with an adrenal adenoma. The left adrenal gland was normal. The patient underwent a laparoscopic adrenalectomy without complications. A 3.0 x 2.5 x 2.5-cm mass was resected. Pathological examination confirmed an adrenal adenoma. The patient was discharged with prednisone temporary replacement.

Five months after the adrenalectomy, the patient developed generalized arthralgias, malaise, and dry cough. Additionally, some erythematous nodular lesions on the skin of the lower extremities were present. A chest X-ray was suggestive of interstitial pneumonitis. A chest CT showed multiple enlarged mediastinal lymph nodes occupying the perivascular space. A ground-glass pattern was present in a segment of the right superior lung lobe, apical and posterior segments of the right inferior lung lobe and posterior segments of the left inferior lung lobe. Additionally, there was bilateral pleural effusion, mainly on the right side. Fibrobroncoscopy was performed, tuberculosis, fungal infection, and malignancy were ruled out by Ziehl-Neelsen staining (ZN), methenamine silver staining, and cytology, respectively.

During endocrinology follow-up, parathyroid hormone (PTH)-independent hypercalcemia (12.7 reference value 8.7-10.4 mg/dL) and hypercalciuria (412 mg/24 hours, reference value 100-200 mg/24 hours) were found. The patient required mediastinoscopy and biopsy of the enlarged lymph nodes. Fungi and acid-alcohol-resistant bacilli infection were ruled out. Due to the presence of a non-necrotizing granulomatous lymphadenopathy, a diagnosis of sarcoidosis was made. The patient was started on oral prednisolone 40 mg per day (0.7 mg per kg per day), azathioprine 100 mg per day and a calcium/vitamin D supplement. Ophthalmic sarcoidosis was ruled out. Follow-up at 13 months showed no signs of recurrence of sarcoidosis or Cushing’s syndrome. The patient remained well with prednisolone treatment, and her symptoms of sarcoidosis did not reappear. The prednisolone dosage was kept unchanged.

## Discussion

Surgical treatment of adrenal Cushing’s syndrome leads to the complete resolution of hypercortisolism, including the associated immunosuppression responsible for the secondary infections that are common in these patients. The cure of Cushing’s syndrome leads to the recovery of the immune system and in some patients triggers autoimmune diseases or unmasks underlying inflammatory conditions [2,3].

In our case, a patient with adrenal Cushing’s syndrome presented after surgical resection of an adrenal adenoma with features highly suggestive of sarcoidosis. The patient presented with arthralgias, cough, mediastinal lymphadenopathy, hypercalcemia, and erythema nodosum. These features are highly suggestive of sarcoidosis [2]. The differential diagnosis of sarcoidosis is tuberculosis and malignancy, therefore, histological confirmation was needed. The finding of mediastinal lymphadenopathy associated with a non-necrotizing granulomatous process supported the diagnosis of sarcoidosis. Tuberculosis and malignancy were ruled out through immunohistochemistry, specific stains and mycobacterium cultures. The finding of erythema nodosum was also highly suggestive of sarcoidosis. It is interesting to note that the diagnosis of sarcoidosis is a challenge because only one-third of patients present with skin findings. Studies suggest that cutaneous sarcoidosis is more common in women. Our patient presented with arthralgias, and it is important to note that acute arthritis occurs in 10% to 40% of patients with sarcoidosis, mainly in the initial phases of the disease. Arthralgias may be the first manifestation of the disease or may be a part of Lofgren’s syndrome (which comprises hilar adenopathy, acute polyarthritis, and erythema nodosum). Usually, the joint symptoms are symmetric and oligoarticular [4].

In this case, the excessive elevation of glucocorticoid levels in Cushing’s syndrome apparently masked the sarcoidosis, and sarcoidosis symptoms manifested only after Cushing’s syndrome was treated by laparoscopic adrenalectomy. New-onset disease or exacerbations of other autoimmune diseases, including rheumatoid and seronegative arthritis, autoimmune thyroiditis, sclerosing pancreatocholangitis, retinal vasculitis, celiac disease, systemic lupus erythematosus and dermatological diseases, such as atopic dermatitis, vitiligo, psoriasis, and pemphigus, have also been reported after Cushing’s syndrome treatment [1,3]. Underlying pathophysiologic mechanisms for sarcoidosis are yet to be determined; as a result, the disease has no known cause. However, experts postulate that sarcoidosis may be an antigenic immune response to mycobacterium infection, given the similarities in clinical presentation and histopathology to tuberculosis.

The endocrine system has an important effect on the immune system because of the hormones and neuropeptides secreted by immune cells. Glucocorticoids (GC) are the major endogenous mechanism by which inflammatory response genes are suppressed, and that is why oral corticosteroids are the first line treatment for sarcoidosis. The pathogenesis of sarcoidosis relies on the interaction of CD4+ T cells with a variety of antigen-presenting cells to trigger granuloma formation. GC have inhibitory effects on a broad range of T-cell and B-cell responses and exhibit potent suppressive effects on the effects or functions of phagocytes. The effect on lymphocytes is mainly directed at T-cell lymphocyte proliferation through the inhibition of IL-1 production by monocyte-macrophages and the inhibition of IL-2 by activated T-cells. The ratio of circulating CD4+/CD8+ lymphocytes is also decreased in Cushing’s syndrome. GC inhibit the production of pro-inflammatory cytokines and metalloproteinases and stimulate the production of anti-inflammatory cytokines, shifting the Th1/Th2 balance toward the Th2 phenotype, which stimulates humoral immunity. This makes them effective in controlling a wide range of inflammatory and autoimmune diseases, such as sarcoidosis.

In addition to the present case, there have been few case reports describing the development of sarcoidosis after treatment of Cushing’s syndrome. Recently, a case report stated that there were only five cases from 1970 to 2014 [2]. A new review of the literature was performed in Medline. This review used the terms “sarcoidosis” and “Cushing” and did not impose language restrictions. Twenty-three manuscripts were found, 12 narrative reviews or unrelated studies, one case report in Japanese, and one unavailable reference were excluded in this manuscript. We found 10 publications including similar case reports published between 1967 and 2017. Two cases were excluded due to space limitations. Main clinical features from the literature review and our case are given in Table 2.

**Table 2:** Similar cases of sarcoidosis following Cushing’s syndrome reported in the literature. F: Female; M: Male; PA: Pituitary adenoma; PAND: Primary adrenocortical nodular dysplasia; AA: Adrenal adenoma; NR: Not reported.

Authors	Age	Gender	Etiology Cushing	Latency (Months)	Clinical Features
Diernaes et al. [2]	46	F	PA	3	Erythema nodosum and bilateral hilar lymphadenopathy
Akama et al. [5]	23	M	PAND	NR	Iridocyclitis and bilateral hilar lymphadenopathy
Steuer et al. [6]	42	F	AA	1.5	Polyarthralgia and subcutaneous nodules
Schaefer et al. [7] A	32	F	AA	1	Subcutaneous nodules, stiffness, arthralgia, left hilar lymphadenopathy
Schaefer et al. [7] B	43	M	PA	1	Subcutaneous nodules, mediastinal and bilateral hilar lymphadenopathy
Fichtel et al. [8]	42	F	PA	3	Subcutaneous nodules
Takenaka et al. [9]	32	F	PA	72	Subcutaneous nodules, mediastinal and bilateral hilar lymphadenopathy, erythema nodosum
Marzano et al. [10]	33	F	PA	2	Subcutaneous nodules, bilateral hilar lymphadenopathy
da Mota et al. [3]	27	M	PA	12	Subcutaneous nodules, mediastinal and bilateral hilar lymphadenopathy
This report	45	F	AA	5	Hypercalcemia, arthralgia, subcutaneous nodules and mediastinal lymphadenopathy

Finally, we postulate that the high cortisol level in Cushing’s syndrome generates immunosuppression, thereby predisposing patients to infection and potentially masking underlying autoimmune diseases. Once Cushing’s syndrome is treated, recovery of patients’ immune status may reveal sarcoidosis or other immune diseases.

## Conclusions

We described a rare case of sarcoidosis after the remission of Cushing’s syndrome to draw attention to the fact that high cortisol levels can lead to immunosuppression; as a result, after the treatment of Cushing's syndrome, the recovery of immune status may unmask inflammatory syndromes such as sarcoidosis. It is important to consider the possibility that autoimmune and corticosteroid-responsive diseases may develop after Cushing’s syndrome remission.
